# Effect of Graphene Nanoplatelets on the Physical and Antimicrobial Properties of Biopolymer-Based Nanocomposites

**DOI:** 10.3390/ma9050351

**Published:** 2016-05-09

**Authors:** Roberto Scaffaro, Luigi Botta, Andrea Maio, Maria Chiara Mistretta, Francesco Paolo La Mantia

**Affiliations:** Dipartimento di Ingegneria Civile, Ambientale, Aerospaziale, dei Materiali, Università di Palermo, UdR INSTM di Palermo, Viale delle Scienze, Palermo 90128, Italy; luigi.botta@unipa.it (L.B.); andrea.maio@unipa.it (A.M.); mariachiara.mistretta@unipa.it (M.C.M.); francescopaolo.lamantia@unipa.it (F.P.L.M.)

**Keywords:** nanocomposites, graphene nanoplatelets (GnPs), poly(lactic acid) (PLA), antimicrobial activity, drug release, ciprofloxacin

## Abstract

In this work, biopolymer-based nanocomposites with antimicrobial properties were prepared via melt-compounding. In particular, graphene nanoplatelets (GnPs) as fillers and an antibiotic, *i.e.*, ciprofloxacin (CFX), as biocide were incorporated in a commercial biodegradable polymer blend of poly(lactic acid) (PLA) and a copolyester (BioFlex^®^). The prepared materials were characterized by scanning electron microscopy (SEM), and rheological and mechanical measurements. Moreover, the effect of GnPs on the antimicrobial properties and release kinetics of CFX was evaluated. The results indicated that the incorporation of GnPs increased the stiffness of the biopolymeric matrix and allowed for the tuning of the release of CFX without hindering the antimicrobial activity of the obtained materials.

## 1. Introduction

Biopolymers are an alternative to oil-based synthetic polymers since they are renewable and do not contribute to environmental pollution being biodegradable, and they are currently used in several applications [1,2,3,4,5,6]. Nevertheless, a broad use of biopolymers is often restricted by the necessity of improving some functional properties such as mechanical and barrier properties. Therefore, intense efforts have been made to improve their physical properties in order to enhance the commercial potential of biopolymers such as poly(lactic acid) (PLA) [7,8,9,10,11].

An effective way to improve the properties of biopolymers is to incorporate nano-sized reinforcements in the matrix [12,13,14,15,16]. Indeed, it is well known that nanocomposites, *i.e.*, polymers filled with particles having at least one dimension in the nanoscale range, show unique properties because of the peculiar increase of the matrix-filler interface [17]. Various nano-sized fillers, such as layered silicates, metal, polyhedral oligomeric silsesquioxane, carbon nanomaterials, and silica nanoparticles, are being developed and extensively studied in the field of polymer nanocomposites. Among the above materials, graphene, a flat monolayer of sp^2−^ bonded carbon atoms, is very promising due to its unique characteristics such as high electronic conductivity, large specific surface area, and high mechanical strength. Graphene combines a layered structure of clays with superior mechanical and thermal properties of carbon nanotubes, which can provide excellent functional property enhancements.

Recently, many studies have been conducted to provide biopolymers with antimicrobial properties that might encourage their use in the field of active food packaging as well as in specific biomedical applications [18,19,20,21,22,23].

Providing a polymeric device with antibacterial properties can be reached by different routes, with or without the modification of the polymer structure. The incorporation of antimicrobials, or other molecules, into a polymer matrix via melt-processing is a method that has been widely adopted in the recent past since it has the advantage of using equipment commonly and already used to process thermoplastic materials [2,3,24,25,26,27,28,29,30,31]. This method, moreover, ensures large production volumes and solventless systems with obvious positive economic and environmental implications.

The effectiveness of the antimicrobial activity over time is mainly determined by the release rate of the antimicrobial compounds. Release kinetics that are either too slow or too fast must be avoided since the former means that microbial growth is not sufficiently inhibited and the latter means that inhibition will not be sustained over time. The rate of release depends on different factors, *i.e.*, the type of polymeric matrix, the preparation method, the environmental conditions, the interactions between the antimicrobial, and the matrix. In this regard, nanoparticles can potentially be used to control the release of antimicrobial agents as reported in the scientific literature [17,32].

The aim of this work was to prepare and characterize biopolymer-based nanocomposites with antimicrobial properties suitable for medical device packaging. In particular, graphene nanoplatelets (GnPs) as fillers and ciprofloxacin (CFX) as biocide were incorporated via melt-compounding in a commercial biodegradable polymer blend (BioFlex^®^) based on PLA. CFX is a wide-spectrum antibiotic belonging to the fluoroquinolone family, active against both Gram-negative and Gram-positive strains. Its spectrum of activity includes most strains of bacterial pathogens responsible for respiratory, urinary tract, gastrointestinal, and abdominal infections.

The rheological, mechanical, and antimicrobial properties of the obtained nanocomposites were evaluated. In particular, the influence of GnPs on the antimicrobial properties and release kinetics was studied.

## 2. Materials and Methods

### 2.1. Materials

The polymer matrix used in this work was a sample of a biodegradable polymer blend of proprietary composition (Bioflex), trade name Bio-Flex^®^ F2110, supplied by FKUR (Willich, Germany). It is based on PLA and a biodegradable copolyester and contains some additives.

Graphene nanoplatelets (GnPs), trade name xGnP^®^, Grade C, were supplied by XG Sciences Inc., Lansing, MI, USA. Each particle consists of several sheet of graphene with an average thickness of approximately 10–20 nm, average diameter between 1 and 2 µm, and a specific surface area of about 750 m^2^/g.

Ciprofloxacin (CFX, chemical formula: C_17_H_18_FN_3_O_3_, T_m_ = 253–257 °C) was supplied by Sigma Aldrich (St. Louis, MO, USA) and used as received without further purification. Its water solubility is 80 mg/L at 30 °C and 120 mg/L at 40 °C [33].

### 2.2. Preparation of Nanocomposites and the Incorporation of CFX

GnPs were added to Bioflex at 1 wt % and 5 wt % at the solid state, and the mixtures have then melt-compounded in a batch mixer (Brabender PLE330, Duisburg, Germany) at 170 °C and a rotational speed of 60 rpm for 5 min. For comparison, a pristine polymer-blend matrix was processed under the same conditions. Before processing, Bioflex and GnPs were dried under vacuum at 90 °C for 4 h and at 120 °C overnight, respectively.

The incorporation of CFX was achieved via melt-compounding using the same batch mixer described above at the same processing conditions, *i.e.*, 170 °C and a rotational speed of 60 rpm. In detail, both the polymer and the GnPs were first fed to the mixer and compounded for 4 min. Thereafter, the CFX was added and the blend was processed for no longer than 1 min in order to avoid eventual degradation phenomena of the additive. Both CFX and GnPs were added to the polymeric matrix at 5% (w/w). For comparison, Bioflex, incorporating only CFX at 5% (w/w), was processed under the same conditions. In Table 1, the composition of all the samples and their identification codes are reported.

Films were prepared by compression-molding using a laboratory press (Carver, Wabash, IN, USA). The material was preventively ground, placed in a mold between two Teflon sheets, and pressed at 170 °C and 100 bar for about 2 min to obtain a 200-μm-thick film.

### 2.3. Characterizations

The morphology of all the materials, including neat GnPs and neat CFX, was analyzed by scanning electron microscopy (SEM; Quanta 200 ESEM, FEI, Hillsboro, OR, USA). In particular, both the GnPs powder and the CFX powder were directly glued onto a sample holder, whereas the polymeric samples were fractured under liquid nitrogen and then glued onto a sample holder. All the samples were sputter-coated with a thin layer of gold to avoid electrostatic charging under the electron beam.

The rheological characterization was performed using a plate–plate rotational rheometer (HAAKE MARS, Thermo Scientific, Waltham, MA, USA), operating at 170 °C on samples obtained by compression-molding. The instrument has been set to operate in the frequency sweep mode in the range 0.1–500 rad/s with a strain of 5%. Before testing, the samples were dried for 4 h under vacuum at 90 °C.

Tensile mechanical measurements were carried out using a dynamometer (Instron model 3365, High Wycombe, UK) on rectangular shaped specimens (10 × 90 mm) cut off from films prepared by compression-molding as described above. The grip distance was 30 mm, and the crosshead speed was 5 mm/min.

The antimicrobial activity of the materials was determined by the agar diffusion method evaluating the presence of inhibition zones. In particular, *Micrococcus luteus* ATCC 10240 (ATCC, Manassas, VA, USA) was used as a tester strain in order to study antimicrobial property of the prepared materials. A bacterial suspension of ~10^9^ colony forming units (CFU) was inoculated into 5 mL of lysogeny broth (LB)-soft agar to obtain a uniform bacterial overlay on LB-agar plates. Circular samples (diameter 12 mm) containing CFX at 5% (w/w) were placed over a bacterial tester overlay. Samples without CFX, *i.e.*, BIO and BIO/GnP-5, were used as controls. Bacterial growth inhibition halos were observed after overnight incubation at 37 °C.

A series of CFX solutions of distilled water containing 0.1–5 mg/L of CFX was used to obtain a calibration curve correlating the absorbance peak intensity and the CFX concentration using a UV/vis spectrophotometer (model UVPC 2401, Shimadzu Italia s.r.l., Milan, Italy). In the concentration range here investigated, the calibration curve was found to be a line. The maximum absorbance peak of CFX was detected at 276 nm. The release of CFX from the films was investigated by immersing a pre-weighed sample in 10 mL of distilled water stored at 37 °C. At specific time intervals, for 6 weeks, the absorbance peak intensity at 276 nm of the storage solutions was measured and converted to the quantities of CFX released based on the calibration line previously calculated. After each measurement, the samples were immersed in 10 mL of fresh distilled water and the cumulative release of CFX here reported was calculated by sequentially adding the CFX released after each step. Each measurement was performed in triplicate.

## 3. Results and Discussion

### 3.1. Properties of Nanocomposites

**Figure 1:**
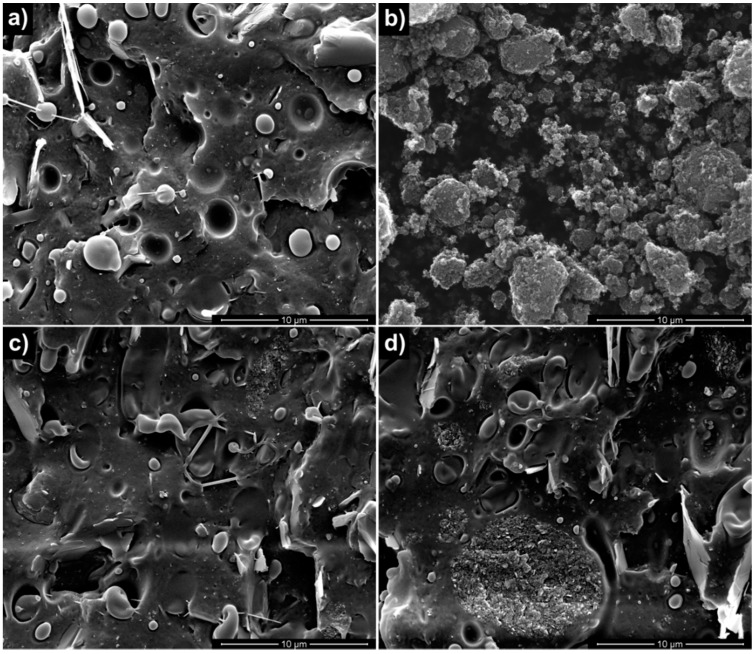
SEM micrographs of: (**a**) BIO; (**b**) neat GnP; (**c**) BIO/GnP-1; (**d**) BIO/GnP-5.

SEM micrographs of the neat polymeric matrix, of the GnPs, and of the nanocomposite systems are reported in Figure 1. The surface fracture of neat BIO (Figure 1a) exhibited the typical morphology of polymer blends showing a poor interfacial adhesion, evidenced by the presence of voids due to the detachment of the copolyester phase from the PLA matrix during the sample breaking. The SEM micrograph of neat GnPs powder (Figure 1b) revealed irregular aggregates having different dimensions. BIO/GnP-1 (Figure 1c) showed a fairly good dispersion of the GnPs and a good adhesion between the matrix and the filler. As expected, upon increasing the filler concentration, the aggregates visible in the fracture section increased, although a good adhesion was still evident (Figure 1d).

**Figure 2:**
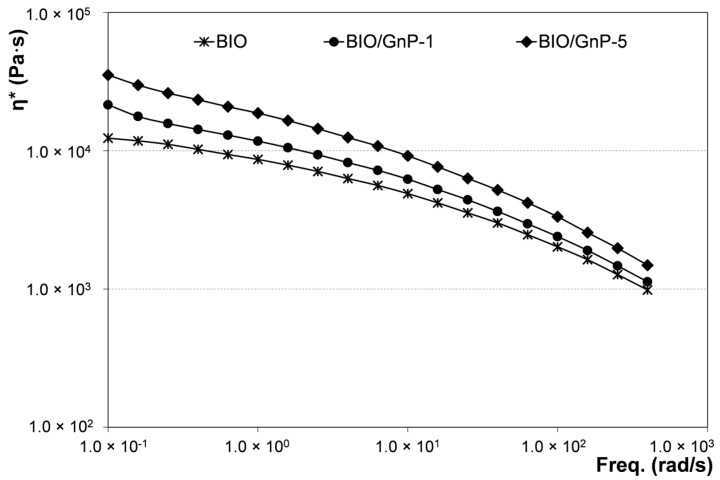
Complex viscosity as a function of frequency of neat BIO and related nanocomposites.

The influence of GnPs on the rheological behavior of the obtained systems was evaluated. The complex viscosity values as a function of frequency are reported in Figure 2 for neat BIO and for the related nanocomposites. The viscosity of the samples filled with GnPs was higher than that of the neat matrix, and it increased with increasing the filler content. Moreover, both BIO/GnP-1 and BIO/GnP-5 exhibited a more pronounced non-Newtonian behavior if compared with the unfilled polymer. At high frequencies, the flow curves of the nanocomposite samples and of the unfilled matrix were much closer among them, although the viscosity values of the nanocomposites remained higher than that of the neat BIO. This rheological behavior is reported in the scientific literature as a typical behavior shown by several nanocomposite systems including polymer/clay nanocomposites [34,35] and polymer/GnPs nanocomposites [36,37]. It is generally attributed to a strong interaction between the dispersed filler and the matrix that restricts the polymer chain movements.

In Table 2, the elastic modulus (E), the tensile strength (TS), and the elongation at break (EB) of neat BIO and of the nanocomposite systems are reported. The adding of GnPs provoked an increase of the rigidity of the biodegradable matrix. In particular, the modulus increased on increasing the amount of filler and BIO/GnP-5 showed a tensile modulus about 40% higher than that of neat BIO. Tensile strength and elongation at break decreased in the presence of the nanoparticles. In detail, the decrease of these properties was very slight for BIO/GnP-1, whereas adding the 5% of GnPs led to a higher decrement, especially of the EB. The decrease of the tensile strength can be considered a consequence of the reduction of the elongation at break that was likely due to the presence of some GnPs aggregates, as shown by SEM micrographs. However, the reduction is quite low and does not compromise the use of the material.

### 3.2. Properties of Antimicrobial Nanocomposites

**Figure 3:**
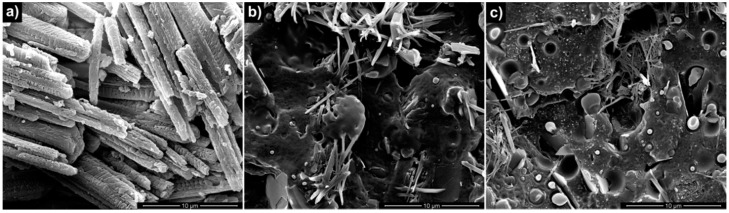
SEM micrographs of: (**a**) neat CFX; (**b**) BIO/CFX; (**c**) BIO/GnP-5/CFX.

Following the results described above regarding the effect of GnP amount on properties of nanocomposites, CFX was incorporated in the nanocomposite system containing the 5% of GnPs. For comparison, BIO, incorporating only the CFX, was prepared. In Figure 3, the SEM micrographs of neat CFX, BIO/CFX, and BIO/GnP-5/CFX are reported. The micrograph of neat CFX powder (Figure 3a) showed that CFX is visible under the form of irregular crystalline aggregates formed by needle-like crystals. These aggregates were clearly visible in the cross section of the films containing CFX as shown by SEM micrographs reported in Figure 3b,c. In particular, the CFX was well dispersed both in the neat polymeric matrix (Figure 3b) and in the nanocomposite system (Figure 3c) in which both the CFX and the GnPs were clearly visible. Moreover, in both cases, it is evident that the dimensions of the CFX aggregates are smaller than the initial ones. This change in size can be attributed to the melt-compounding that caused a disaggregation of the CFX clusters and the dispersion of the particles into the matrix. Nevertheless, the adhesion level between CFX crystals and the polymer matrix is quite poor.

**Figure 4:**
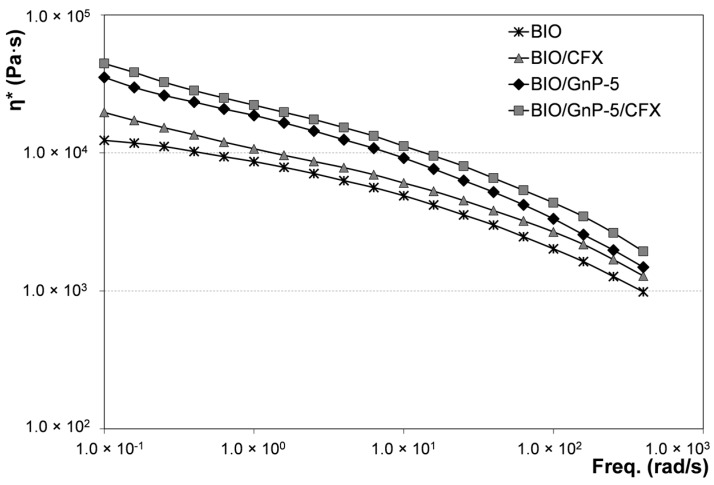
Complex viscosity as a function of frequency of systems incorporating CFX and of the respective systems without antimicrobial additive.

The rheological behavior of the systems incorporating the antibiotic was evaluated and compared with the respective systems without CFX. In Figure 4, the complex viscosity values as a function of frequency of BIO/CFX and BIO/GnP-5/CFX were reported and compared with the flow curves of BIO and BIO/GnP-5 shown above.

The presence of CFX led to a slight rising of the flow curves both of the neat biopolymeric matrix and of the nanocomposite system. Indeed, CFX acted as a micro-filler, causing the light increment of the viscosity in the whole range of investigated frequencies.

In order to verify whether the CFX incorporation caused some modification of the mechanical performance of the materials, tensile tests were performed. Table 3 reports the elastic modulus (E), the tensile strength (TS), and the elongation at break (EB) of all the materials containing CFX. The results showed that, for both systems, *i.e.*, BIO/CFX and BIO/GnP-5/CFX, adding the antimicrobial additive led to a slight increase of E and a decrease of EB. Indeed, CFX acted as a micro-filler, causing, at this concentration, a slight increment of the rigidity and decrement of the ductility.

**Figure 5:**
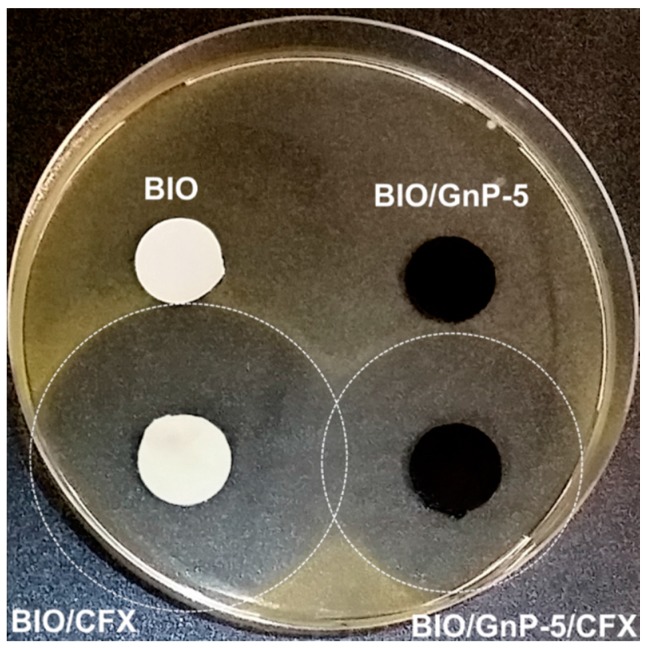
Agar diffusion test performed on *M. luteus* overlay for the materials incorporating CFX and for the respective systems without antimicrobial additive.

To verify that the incorporation of CFX conferred antimicrobial activity to the polymeric systems, agar diffusion tests were performed. A Gram-positive bacterium, *i.e.*, *M. luteus*, was used as tester strain to evaluate the growth inhibition zone around the samples. Figure 5 reports the bacterial inhibition halos observed around the systems incorporating the CFX and, for comparison, the respective systems without antimicrobial.

As expected, both the neat BIO and BIO-GnP-5 showed no antibacterial activity. On the contrary, large bacterial growth inhibition halos were observed around both samples incorporating CFX after an overnight incubation at 37 °C. More specifically, the presence of GnPs led to a reduction of the inhibition zone, *i.e.*, the inhibition halo diameter of BIO/CFX was about 42 mm, whereas BIO/GnP-5/CFX exhibited an inhibition halo diameter of about 35 mm. This result can be explained considering that the antimicrobial properties of the films are dependent on the release of CFX from themselves and that the presence of the GnPs likely influenced the CFX release from the sample.

**Figure 6:**
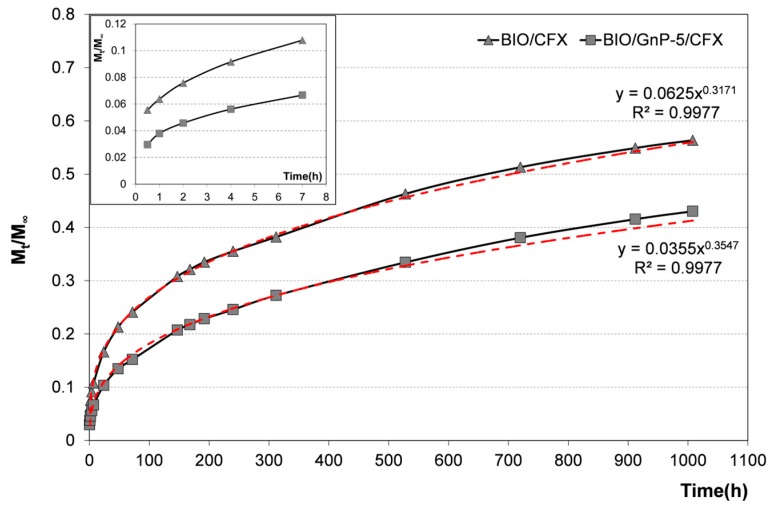
Cumulative CFX release from BIO/CFX and BIO/GnP-5/CFX. In the inset, only the first hours of release are plotted in order to enhance their readability. The power trend-lines fitting the experimental data (red dashed lines) are reported in the graph together with the respective equations and the R^2^ values.

In order to verify this hypothesis, the release kinetics in distilled water at 37 °C was evaluated. In Figure 6, cumulative CFX release as a function of the time from BIO/CFX and BIO/GnP-5/CFX are reported. In particular, the release data were expressed as *M_t_/M_∞_*, where *M_t_* is the cumulative amount of drug released at time t, and *M_∞_* is the cumulative amount of drug released at infinite time (which should be equal to the theoretical absolute amount of drug incorporated within the system at time t = 0).

For both systems, the release of CFX was characterized by an initial burst phase followed by a second phase, which is characterized by a slower release rate. However, according to results of the agar diffusion test, BIO/GnP-5 released a lower amount of CFX during the entire six weeks of immersion. This result can be explained considering that the incorporated GnPs into the polymer matrix was able to create a tortuous pathway, thus slowing down the diffusion of drug molecules through the polymeric matrix, as already reported in the scientific literature for other nanocomposite systems [17,32].

In order to understand the release mechanism, the experimental data were fitted using the well-known power law model:

(1) $$\frac{M_{t}}{M_{\infty }}=kt^{n}$$

where *k* is a kinetic constant related to the properties of the drug delivery system, and *n* is the diffusion exponent that characterizes the release mechanism. In particular, when the value of *n* is ≤0.5, it indicates that the drug release follows the Fickian diffusion mechanism [38,39]. The power trend-lines fitting the experimental data (red dashed lines) are reported in Figure 6 together with the related equations and the R^2^ values. It is worth noting that the power law model well fitted the experimental release data since R^2^ values were very high, *i.e.*, 0.9977 for both systems. The *n* values obtained by the fitting were 0.3171 and 0.3547 for BIO/CFX and BIO/GnP-5/CFX, respectively. This implies that the release of CFX from both systems followed a diffusion-controlled mechanism. The *k* value was generally related to the release kinetics, *i.e.*, a higher *k* value indicates a faster release. As expected, the value of *k* was higher for the release from BIO/CFX in comparison with the *k* value obtained from the release profile of BIO/GnP-5/CFX.

## 4. Conclusions

Biopolymer-based nanocomposites with antimicrobial properties filled with graphene nanoplatelets (GnPs) were prepared via melt-compounding in a batch mixer. An antibiotic, *i.e.*, ciprofloxacin (CFX), was chosen as biocide and incorporated together with GnPs in a commercial biodegradable polymer-blend matrix.

The morphological analysis revealed that the GnPs were well dispersed in the biodegradable matrix, although at the higher concentration some aggregates were visible. The nanocomposites exhibited flow curves higher than that of the neat Bioflex, and the viscosity increased as the filler content was increased. The adding of GnPs improved the stiffness of the matrix—in particular, the elastic modulus increased with an increasing filler amount. The incorporation of GnPs affected the release of CFX without hindering the antimicrobial activity of the obtained materials. In particular, the presence of GnPs led to a slower release of CFX.

## Figures and Tables

**Table 1:** Composition of samples and their codes.

Sample Code	Bio-Flex^®^ F2110 (BIO) (w/w %)	Graphene Nanoplatelets (GnPs) (w/w %)	Ciprofloxacin (CFX) (w/w %)
BIO	100	–	–
BIO/GnP-1	99	1	–
BIO/GnP-5	95	5	–
BIO/CFX	95	–	5
BIO/GnP-5/CFX	90	5	5

**Table 2:** Elastic modulus (E), tensile strength (TS), and elongation at break (EB) of neat BIO and related nanocomposites.

Samples	E (MPa)	TS (MPa)	EB (%)
BIO	140 ± 5.8	11.6 ± 0.5	165 ± 7.2
BIO/GnP-1	168 ± 6.2	11.1 ± 0.4	151 ± 6.1
BIO/GnP-5	195 ± 6.8	10.6 ± 0.6	135 ± 6.9

**Table 3:** Elastic modulus (E), tensile strength (TS), and elongation at break (EB) of BIO/CFX and BIO/GnP-5/CFX.

Samples	E (MPa)	TS (MPa)	EB (%)
BIO/CFX	150 ± 5.1	10.4 ± 0.5	130 ± 9.7
BIO/GnP-5/CFX	206 ± 7.1	10.2 ± 0.6	121 ± 9.9
